# Inhibition of tumor growth by a newly-identified activator for epidermal fatty acid binding protein

**DOI:** 10.18632/oncotarget.3485

**Published:** 2015-03-08

**Authors:** Enyu Rao, Puja Singh, Xiuhong Zhai, Yan Li, Ganqian Zhu, Yuwen Zhang, Jiaqing Hao, Young-In Chi, Rhoderick E. Brown, Margot P. Cleary, Bing Li

**Affiliations:** ^1^ The Hormel Institute, University of Minnesota, Austin, MN, USA

**Keywords:** E-FABP, tumor associated macrophages, interferon β, tumor treatment

## Abstract

Our previous studies have demonstrated that expression of epidermal fatty acid binding protein (E-FABP) in tumor associated macrophages (TAMs) promotes macrophage anti-tumor activity by enhancing IFNβ responses in tumor models. Thus, E-FABP represents a new protective factor in enhancing tumor immune surveillance against tumor development. Herein, we report the compound 5-(benzylamino)-2-(3-methylphenyl)-1,3-oxazole-4-carbonitrile (designated EI-05) as a novel E-FABP activator for inhibition of mammary tumor growth. EI-05 was selected from the ZINC compound library using molecular docking analysis based on the crystal structure of E-FABP. Although EI-05 is unable to bind E-FABP directly, it significantly increases E-FABP expression in macrophages during inflammation. Stimulation of macrophages with EI-05 remarkably enhances lipid droplet formation and IFNβ production, which further promotes the anti-tumor activity of macrophages. Importantly, administering EI-05 *in vivo* significantly inhibits mammary tumor growth in a syngeneic mouse model. Altogether, these results suggest that EI-05 may represent a promising drug candidate for anti-tumor treatment through enhancing E-FABP activity and IFNβ responses in macrophages.

## INTRODUCTION

Tumor initiation and progression are complex processes which depend not only on intrinsic alterations in tumor cells, but also on the extrinsic factors from host immunity. How to enhance tumor immune surveillance remains one of the most active research areas in tumor immunology [1, 2]. Emerging evidence indicates that energetic metabolism of immune cells profoundly impacts their functions and fates [3]. Therefore, investigating the role of distinct metabolic pathways in immune populations and uncovering how these pathways determine their immunologic consequences represent important foci for the control of tumor development.

Fatty acid binding proteins (FABPs) represent a family of small and highly homologous cytoplasmic lipid chaperones that regulate lipid transfer and responses inside cells. Accumulating data have shown that FABPs are central regulators of both metabolic and inflammatory pathways [4, 5]. While most FABP members, such as liver FABP (L-FABP) and intestinal FABP (I-FABP), exhibit tissue-specific patterns of distribution, epidermal FABP (E-FABP) displays a more ubiquitous expression, particularly in immune cells, suggesting an essential role of E-FABP in regulating energy metabolism and functions of immune populations [6, 7]. In our studies focusing on the role of E-FABP in tumor development, we have demonstrated that E-FABP deficiency in mice accelerates mammary tumor growth through impairing IFNβ responses in tumor associated macrophages (TAMs), suggesting E-FABP as a new protective factor in host against tumor development [8]. In addition, we also have reported that E-FABP expression in mice promotes the development of experimental autoimmune encephalomyelitis (EAE) [9]. While effective anti-tumor immune responses require the expression of E-FABP, overactivity of E-FABP in immune cells may instead promote inflammatory autoimmune responses. Thus, our studies suggest that E-FABP functions as an important immune regulator in shaping immune functions in different disease settings, and modulating E-FABP activity may provide an attractive approach for treatment of tumor and autoimmune diseases.

In fact, the use of pharmacological agents to modify FABP function has been proposed in many studies to control inflammatory responses and metabolic syndromes, thus offering a new class of multi-indication therapeutic agents [10–13]. In our efforts to screen for small molecule compounds that may regulate E-FABP activity for potential clinical application, we identified one compound 5-(benzylamino)-2-(3-methylphenyl)-1,3-oxazole-4-carbonitrile (designated EI-05) which enhances E-FABP expression in macrophages and promotes their type I IFNβ responses during inflammation. More importantly, administration of this newly-identified compound significantly inhibited mammary tumor growth *in vivo*.

## RESULTS

### Screening of potential E-FABP modifiers by molecular modeling

**Figure 1:**
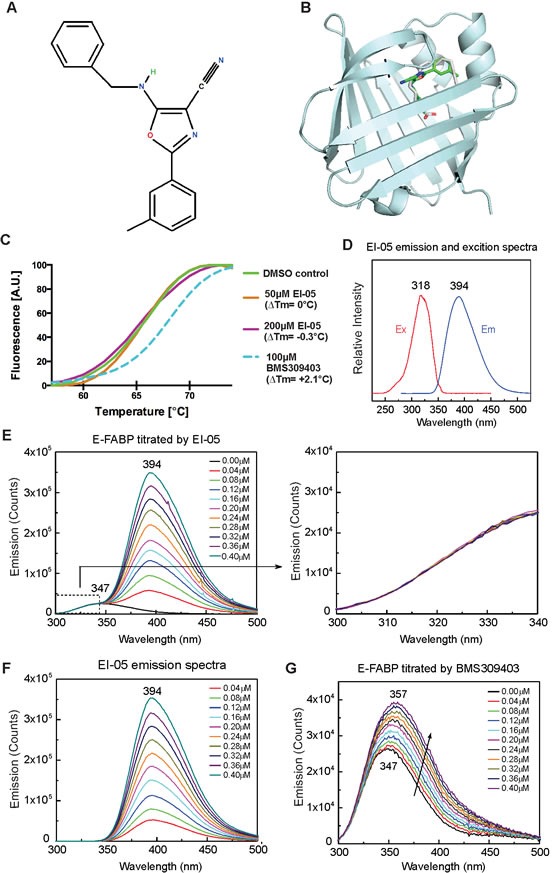
*In-silico* screening of EI-05 (A) Chemical structure of EI-05 (ZINC00467342) (B) The predicted model of EI-05 binding to the lipid-binding pocket of E-FABP. (C) Normalized melting curves depicting enhanced thermal stability of E-FABP by BMS309413 (blue dashed line), but not by EI-05 (orange and purple solid lines). (D) Excitation and emission spectra of EI-05 solved in methanol. (E) Tyr/Trp emission spectra of E-FABP (0.5 μM) in the 300-500 nm range were measured by step-wise addition of indicated concentrations of EI-05. The Try/Trp emission signal between 300-347 nm (dashed box) is enlarged in the right panel. (F) The emission spectra of indicated concentrations of EI-05 in the absence of E-FABP. (G) Tyr/Trp emission of E-FABP (0.5 μM) in the 300-500 nm range was measured by addition of indicated concentrations of BMS309413. Excitation at 270 nm was used for experiments shown in panels E, F and G.

As identification of small molecule inhibitors of FABP has been proven to be effective in treating atherosclerosis and type II diabetes in mouse models [14], we initially intended to screen small molecules that might specifically bind to the lipid-binding pocket of E-FABP using computational docking analysis [15]. Based on the crystal structure of E-FABP (PDB ID 1B56), EI-05 (Figure 1A) was selected as a potential E-FABP partner due to the predicted binding of E-FABP/EI-05 complex shown by molecular docking modeling (Figure 1B). However, when we purified recombinant E-FABP proteins and conducted *in vitro* binding assays, we found that E-FABP did not bind to EI-05, despite good binding to the known inhibitor BMS309403 [16], in thermal shift assays (Figure 1C). As EI-05 exhibited a relatively low excitation signal at 270 nm (Figure 1D), which enabled this wavelength to be used to excite Tyr and Trp in E-FABP, we evaluated the binding of E-FABP/EI-05 by Förster resonance energy transfer based on the spectral overlap of the E-FABP Tyr/Trp emission and EI-05 excitation signals. Step-wise addition of EI-05 to E-FABP did not affect the emission of E-FABP Tyr/Trp (Figure 1E). The strong stepwise increases in EI-05 emission signal at 394 nm, shown in Figure 1E, were nearly the same in the absence of E-FABP (Figure 1F), consistent with no energy transfer. In contrast, positive controls performed with BMS309403 showed the expected dose-dependent increase of E-FABP Tyr/Trp emission (Figure 1G). Thus, although predicted to bind E-FABP by computational modeling, our *in vitro* binding assays clearly indicate that EI-05 has no direct binding to E-FABP.

### EI-05 enhances E-FABP expression in activated macrophages

**Figure 2:**
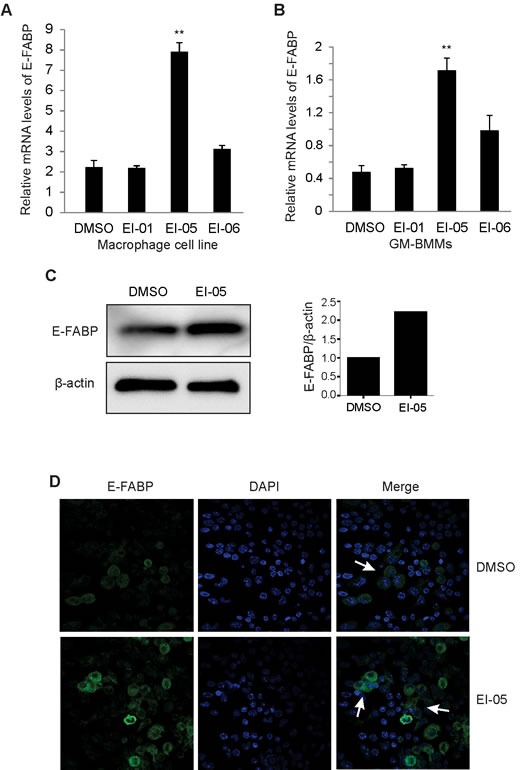
EI-05 enhances E-FABP expression in macrophages Macrophages from a cell line (A) or bone-marrow (GM-BMMs) (B) were activated by LPS (10ng/ml) in the presence of absence of screened E-FABP partners (20 μM) for 24 h *in vitro*, respectively (**, *p* < 0.01 as compared to DMSO group). E-FABP expression was quantified by qPCR. Mice were i.p. injected with EI-05 (10 mg/kg) and vehicle control for 24 h, respectively. PBMCs were measured for E-FABP expression by western blot (C). Peritoneal macrophages were analyzed for E-FABP expression by confocal staining (D).

When we activated a macrophage cell line with LPS in the presence or absence of EI-05 and other potential E-FABP partners identified by computational modeling analysis, we found that EI-05, but not other small molecules, significantly enhanced E-FABP expression in macrophages (Figure 2A). We further investigated the effect of EI-05 on E-FABP expression with primary GM-CSF-induced macrophages derived from mouse bone marrow (GM-BMMs). We demonstrated that E-FABP expression in EI-05-stimulated macrophages was about 4.5 fold higher than that in control groups (Figure 2B). Consistent with these *in vitro* observations, when EI-05 was administered *in vivo*, it was able to greatly enhance E-FABP expression in macrophages of the peripheral blood as shown by western blot analysis (Figure 2C). Confocal microscopy staining further demonstrated that EI-05 treatment greatly enhanced E-FABP expression in the cytoplasm of macrophages (Figure 2D). Thus, these data suggest that EI-05 can activate macrophages through enhanced E-FABP expression in both *in vitro* and *in vivo* conditions.

### EI-05 promotes IFNβ production in macrophages

**Figure 3:**
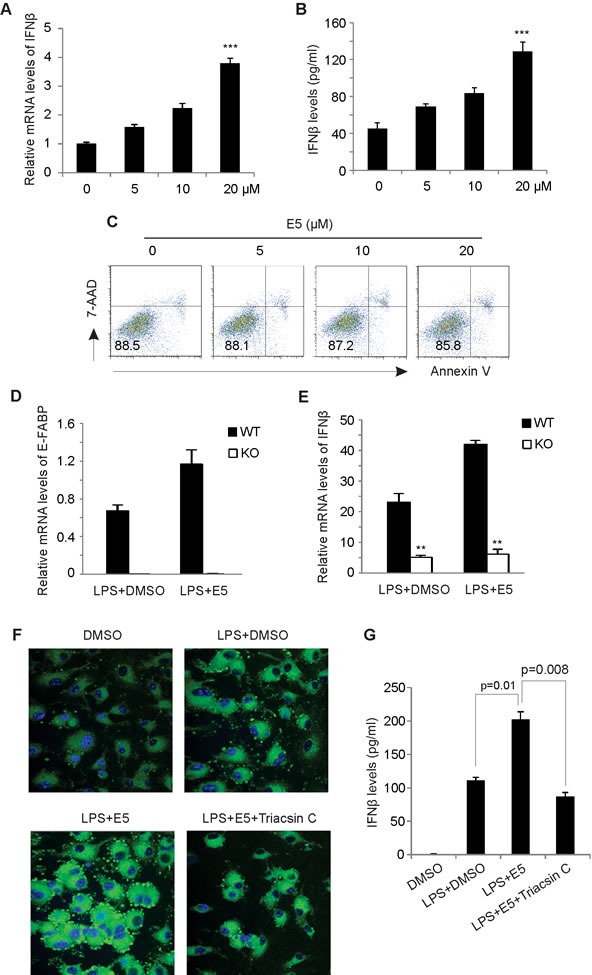
EI-05 treatment promotes IFNβ production in macrophages GM-BMMs were activated by LPS (10 ng/ml) in the presence or absence of indicated concentrations of EI-05 for 24 h. IFNβ expression in macrophages was quantified by qPCR (A). IFNβ protein levels in cultural supernatants were measured by ELISA (B) (***, *p* < 0.001 as compared to the control group). (C) Flow cytometric analysis of 7-AAD and annexin V expression on GM-BMMs treated with indicated concentrations of EI-05 for 24 h. (D-E) E-FABP WT and KO macrophage cell lines were treated activated by LPS (10ng/ml) in the presence of EI-05 or DMSO control for 3h. Expression of E-FABP (D) and IFNβ (E) was analyzed by realtime PCR (**, *p* < 0.01 as compared to WT macrophages). (F) Confocal microscopy analysis of lipid droplet formation (BODIPY) in BM-GMMs with designated treatment with LPS (10 ng/ml), EI-05 (20 μM) or Triacsin C (5 μM). (G) Measurement of IFNβ levels in cultural supernatants of GM-BMMs with indicated treatment.

**Figure 4:**
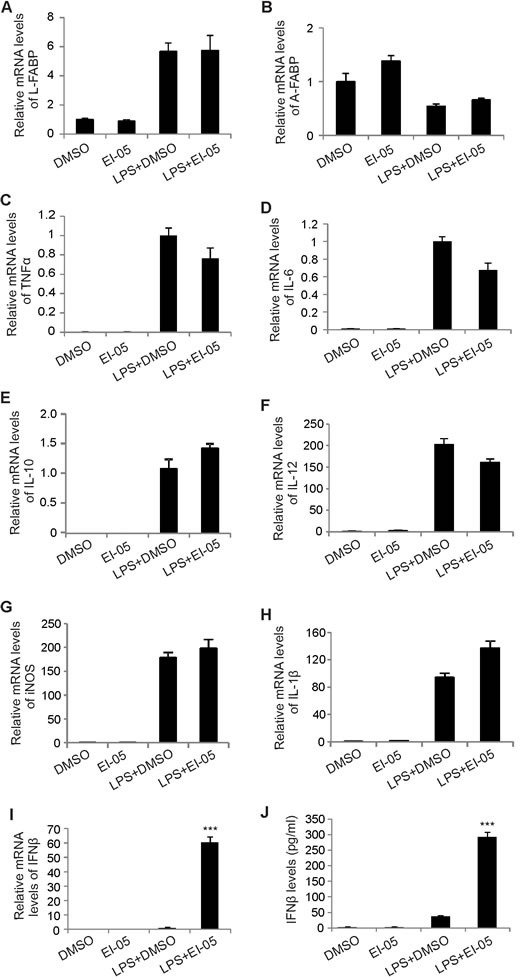
EI-05 treatment has no major impact on other tumor-related molecules in macrophages (A-H) GM-BMMs were cultured with indicated treatment (EI-05, 20 μM; LPS 10 ng/ml) for 24 h. The expression of L-FABP, A-FABP and other tumor-related cytokines were quantified by qPCR. PEMs were cultured with indicated treatment (EI-05, 20 μM; LPS 10 ng/ml) for 24 h. Cells were lyzed for mRNA extraction and PCR analysis of relative RNA levels IFNβ (I). The protein levels of IFNβ in the cultural supernatants were measured by ELISA (J) (***, *p* < 0.001 as compared to the LPS+DMSO group).

As E-FABP expression in TAMs can promote IFNβ responses [8], we next analyzed whether EI-05 treatment impacts IFNβ production in macrophages. Indeed, addition of EI-05 greatly enhanced IFNβ mRNA levels in LPS-activated GM-BMMs (Figure 3A) in a dose-dependent manner. Similarly, IFNβ protein levels in the culture supernatants were also positively elevated in response to increasing concentrations of EI-05 (Figure 3B). As leaking DNA from cellular damage can induce IFNβ production [17], we analyzed the cytotoxicity of EI-05 on macrophages, and demonstrated a minimal impact of EI-05 on macrophage death (Figure 3C), suggesting that a specific effect of IFNβ production was induced by EI-05. When we measured IFNβ production using E-FABP WT and KO macrophages, we found that EI-05 treatment promoted E-FABP and IFNβ production in the WT cells, but not in the E-FABP KO cells (Figure 3D, 3E), indicating an E-FABP-dependent effect for EI-05-induced IFNβ production in macrophages. In our previous studies, we have shown that E-FABP-promoted lipid droplet (LD) formation was positively associated with IFNβ production [8]. It is likely that EI-05 treatment may promote IFNβ production through E-FABP-promoted LD formation. To this end, we measured the impact of EI-05 on LD formation in macrophages. Confocal microscope analysis showed that EI-05 greatly upregulated LD formation in macrophages (Figure 3F). In agreement with our previous results, EI-05-enhanced LD formation and IFNβ production were dramatically inhibited by Tracsin C, a specific LD inhibitor (Figure 3G), further indicating the importance of LDs in mediating the production of IFNβ in macrophages. Of note, EI-05 treatment did not affect the expression of other FABP members, such as L-FABP and A-FABP, and the production of other tumor-related cytokines, such as TNF-α, IL-6, IL-10, IL-12, iNOS, etc (Figure 4A–4H). When we further analyzed the production of IFNβ and other cytokines by peritoneal macrophages (PEMs), we confirmed that that EI-05 also enhanced IFNβ production in these physiologic populations (Figure 4I, 4J). These results indicate that EI-05 treatment greatly promotes E-FABP-mediated IFNβ production in macrophages.

### EI-05 treatment enhances macrophage activation

**Figure 5:**
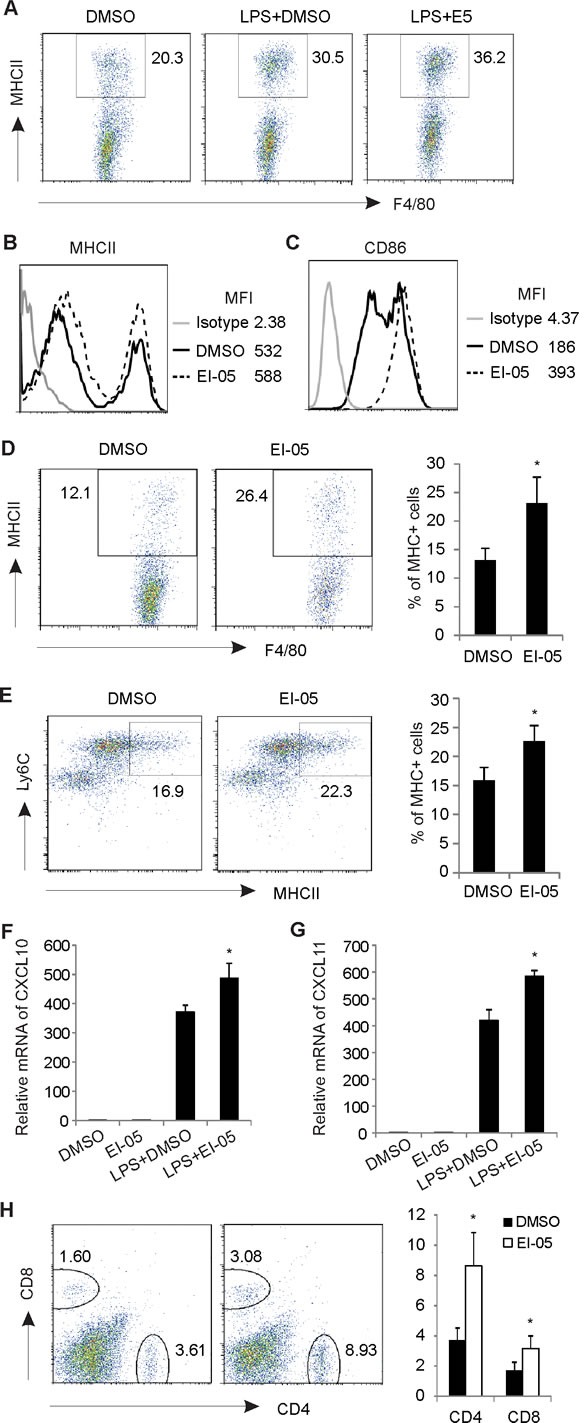
EI-05 promotes macrophage activation and T cell responses Macrophages from peritoneum were collected and stimulated with LPS (10 ng/ml) in the presence or absence of EI-05 (20 μM) for 24 h *in vitro*. (A) Flow cytometric analysis of MHCII^+^ populations in F4/80^+^ macrophages. Mean fluorescent intensity (MFI) of MHCII (B) and CD86 (C) expression on macrophages were analyzed by flow cytometry. Mice were i.p. injected with EI-05 or DMSO, respectively, for 24 h. Flow cytometric analysis of MHCII^+^ populations in F4/80^+^ PEMs (D) and in F4/80^+^ bone marrow macrophages (E). PCR analysis of CXCL10 (F) and CXCL11 (G) expression on macrophages activated by LPS or LPS plus EI-05 for 24 h. (H) Flow cytometric analysis of CD4^+^ and CD8^+^ T cell infiltration in peritoneum of mice administered i.p with either EI-05 or DMSO control (*, *p* < 0.05 as compared to the DMSO group).

Considering the critical role of IFNβ in activating antigen presenting cells [18, 19], we next measured the expression of MHCII and CD86 on macrophages in the presence or absence of EI-05 *in vitro*. As expected, EI-05 treatment not only increased the percentage of MHCII^+^ cells in F4/80^+^ PEMs, but also upregulated the expression intensity of MHCII molecules (Figure 5A, 5B). CD86 expression was also significantly enhanced in response to EI-05 treatment (Figure 5C). To evaluate the relevance of these *in vitro* observations, we intraperitoneally injected mice with either EI-05 or the vehicle and analyzed the phenotypes of macrophages *in vivo*. Consistently, EI-05 administration significantly increased MHCII^+^ populations in F4/80^+^ PEMs as compared to vehicle-treated mice (Figure 5D). In addition, mice treated with EI-05 exhibited elevated MHCII^+^ macrophages in their bone marrow, suggesting a systemic effect of EI-05 (Figure 5E). Moreover, we measured IFN-stimulated chemokines in PEMs and demonstrated that EI-05 treatment greatly enhanced the expression of CXCL10 and CXCL11 (Figure 5F, 5G). Accordingly, there were increased infiltration of CD4^+^ T cells and CD8^+^ T cells in the peritoneum of EI-05-treated mice in comparison to control mice (Figure 5H). Altogether, our data suggest that EI-05 treatment greatly activates macrophages for antigen presentation, and subsequent T cell responses.

### EI-05 treatment reduces mammary tumor growth *in vivo*

**Figure 6:**
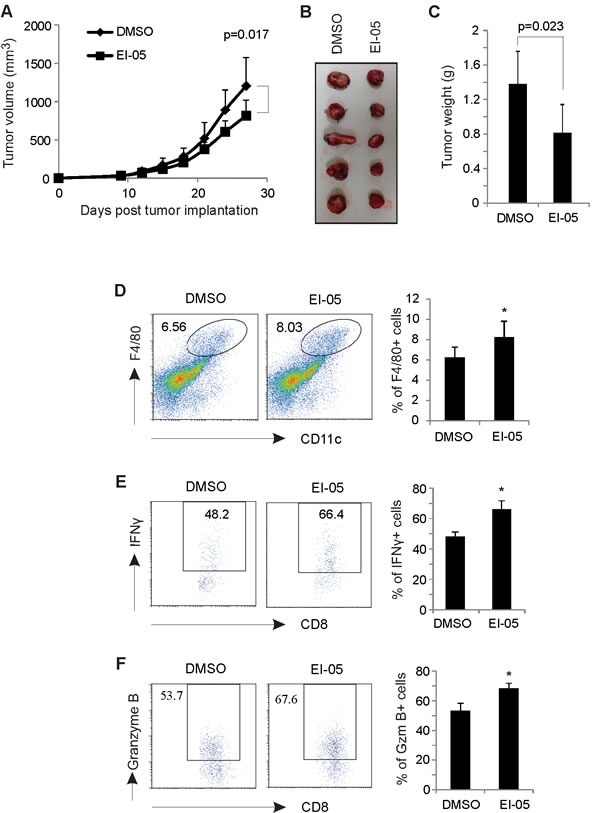
EI-05 treatment inhibits mammary tumor growth E0771 tumor cells (0.5×10^6^/mouse) were orthotopically injected into the mammary fat pad of C57B/C mice (n=20). Mice were randomly divided into two groups (n=10/group) and i.p. administered with either EI-05 (10 mg/kg) or vehicle DMSO control every other day. Tumors were measured at 3-day intervals and tumor growth curve were shown in panel (A). Representative tumor pictures in each group on day 27 after tumor injection were shown in panel (B). (C) Average tumor weights were measured on day 27 after mice were euthanized. (D) Flow cytometric analysis of tumor infiltrated F4/80^+^CD11c^+^ macrophages in EI-05- or DMSO-treated mice. Intracellular staining of the production of IFNγ (E) and Granzyme B (F) in tumor infiltrated CD8^+^ T cells from EI-05- or DMSO-treated mice (*, *p* < 0.05 as compared to the DMSO group).

To investigate the therapeutic efficacy of EI-05 for tumor treatment, we orthotopically inoculated mouse E0771 mammary tumor cells into the mammary fat pat of mice and measured the tumor growth in EI-05- or vehicle-treated mice. Very strikingly, tumor growth in EI-05-treated mice was significantly decreased in volume (about 40%) as compared to vehicle-treated group (Figure 6A). Accordingly, tumors in EI-05 treated mice exhibited reduced size and weight than those in control mice (Figure 6B, 6C). Interestingly, EI-05 treatment greatly increased the percentage of F4/80^+^CD11c^+^ TAMs in tumors (Figure 6D). It is worth noting that EI-05 administration also remarkably enhanced the production of IFNγ and Granzyme B by tumor-infiltrated CD8^+^ T cells (Figure 6E, 6F). Thus, these results suggest that EI-05 administration is able to inhibit mammary tumor growth, thus representing a novel drug candidate for anti-tumor treatment.

## DISCUSSION

We have previously demonstrated that E-FABP expression in TAMs promotes their anti-tumor phenotype through enhanced IFNβ responses in mammary tumor models [8]. This study suggests E-FABP as a new protective factor that can enhance tumor immunosurveillance. Thus, identification of new chemicals or dietary factors which specifically upregulate E-FABP expression in macrophages may provide novel strategies for prevention and treatment of cancer. In this follow-up study, we identified EI-05 as a new E-FABP activator for inhibition of mammary tumor growth in a syngeneic mouse model.

To identify small molecules which may bind E-FABP and inhibit its activity and be used for treatment of autoimmune diseases, we screened more than one million compounds from the ZINC library using *in-silico* docking analysis with Glide XP [15, 20]. Based on the score ranking and the molecular docking model against the E-FABP protein structure, EI-05 and several other compounds attracted our attention due to their potential binding to the lipid-binding pocket of E-FABP. However, when we conducted *in vitro* binding assays to detect protein-ligand interactions using various independent methods, such as fluorescent measurements, thermal shift assays and bead pull-down assays, none of the data showed any positive evidence of effective EI-05/E-FABP interactions. Because the positive binding of BMS309403 and other compounds to E-FABP were clearly observed in our assays, we were convinced that EI-05 actually does not bind to E-FABP (Figure 1). However, when we performed biological experiments with EI-05, we found that E-FABP is able to specifically enhance E-FABP expression in macrophages from different origins (Figure 2). Since the molecular mechanisms by which E-FABP is regulated remain largely unknown, it warrants further investigation to determine how EI-05 enhances E-FABP expression in macrophages.

Given that E-FABP upregulation in TAMs promotes IFNβ production and signaling to promote macrophage anti-tumor activity [8], we further analyzed whether EI-05 enhances IFNβ responses in macrophages. In fact, EI-05 treatment greatly enhances IFNβ production in both bone marrow-derived macrophages and PEMs (Figures 3, 4). In agreement with our previous studies, EI-05 markedly promotes LD formation in macrophages, which facilitates the enhanced production of IFNβ. We noticed that LD inhibition does not completely inhibit EI-05-enhanced production of IFNβ, thus, it is possible that EI-05 may also utilize other pathways to regulate IFNβ production. It has been shown that IFNβ production can be regulated by PPARγ in TLR3- and TLR4-stimulated macrophages [21]. We observed that EI-05 treatment promotes nuclear translocation of PPARγ and PPAR β/δ in macrophages (data not shown). Considering E-FABP as a target gene for PPARβ/δ [22], it is likely that EI-05 may regulate the transactivation of PPARs, thereby contributing to IFNβ production in macrophages.

Due to the critical role of type I IFNs in regulating innate and adaptive immunity, IFNα/β have been used in treatment of different types of tumors [23–25]. Unlike IFNγ which acts directly on tumor cells, IFNα/β exerts its anti-tumor activity mainly through acting on immune populations, such as CD11c^+^ APCs, CD8^+^ T cells, etc [19, 26]. For example, type I IFN endogenously produced by innate cells can bridge adaptive immune responses by upregulating MHC molecule expression on APCs, and enhancing Granzyme B-mediated tumor cytotoxicity of tumor infiltrated CD8^+^ T cells, all of which are supported by our data (Figures 5, 6). However, it should be pointed out that exogenously administered IFNα/β is often accompanied by many side effects, which extensively limit its clinical use. In addition, the pleiotropism and redundancy of cytokine signaling make their use for immunotherapy a great challenge *in vivo.* It has been reported that exposure to exogenous IFNβ can decrease the expression of IFNGR1 and inhibit IFNγ-induced activation of macrophages [27]. Thus, the efficacy of direct application of exogenous IFNβ is very mixed and can be compromised due to the cross-regulation between type I and type II IFNs.

Since EI-05 can enhance E-FABP expression and endogenous IFNβ production by macrophages, it is logical to speculate that administering of EI-05 may be beneficial for anti-tumor therapy. To prove our concept, we first tested the toxicity of EI-05 application *in vivo* at the dose of 5 mg/kg/mice, 10 mg/kg/mice and 20 mg/kg/mice, respectively, based on other small molecules used *in vivo* [14]. All tested mice showed no toxic responses to EI-05 treatment regarding mouse weight, numbers of PBMCs, immune cell phenotype and cytokine production (data not shown), so we tested the efficacy of EI-05 using the middle dose at 10 mg/kg for tumor treatment with a syngeneic mouse mammary tumor model. Indeed, we demonstrated that EI-05 has great therapeutic efficacy in suppressing mammary tumor growth. Although more studies are under investigation to optimize the routes of administration and to test its efficacy in other tumors, EI-05 represents a promising drug candidate for anti-cancer treatment in many aspects. 1) It is clear that cell metabolism profoundly impacts their function [28, 29]. As the predominant FABP member in immune cells, E-FABP expression is critical in coordinating metabolism and function in immune cells [30]. Thus, enhancing E-FABP expression by EI-05 will facilitate their anti-tumor activity. 2) Due to the dual roles of IFNα/β in anti-tumor effect and pro-autoimmune responses [23], it is very hard to control the right dosage of IFNβ when it is administered exogenously. Instead, administering of EI-05 will potentially reduce the side effects of exogenous IFNβ in that EI-05-enhanced IFNβ will be coordinately produced by host immune cells. 3) EI-05 itself shows a minimal toxic effect on either cultured cells or on mice when administered *in vivo*. Therefore, EI-05 has great potential clinical application.

In summary, we have identified EI-05 as a new activator for E-FABP. It not only enhances E-FABP expression in macrophages but also promotes their IFNβ production and responses. More importantly, administering EI-05 *in vivo* significantly suppresses mammary tumor growth. Thus, enhancing E-FABP activity by EI-05 may provide a new strategy for cancer prevention and treatment.

## METHODS

### *In silico* screening for E-FABP modifiers

Based on the crystal structure of E-FABP (PDB ID 1B56), *in-silico* screening was conducted using the program Glide v5.7 [15]. Flexible docking was performed with the standard precision (SP) mode. More than one million compounds from the ZINC database were screened for potential E-FABP modifiers. The top-ranked compounds were clustered into different classes based on similarity of chemical structures. Compound EI-05 was selected due to its potential interaction with lipid binding pocket of E-FABP. EI-05 was purchased from Interbio Screen Ltd for biological experimental tests.

### *In vitro* binding assays

Recombinant E-FABP was expressed in BL21 (DE3) and purified using Ni-NTA resin followed by the cleavage of His-tag and ion-exchange chromatography. Endogenous lipids bound to E-FABP were removed by running through the Lipidex-1000 column. Binding of EI-05/E-FABP was determined by two independent assays, in addition to a compound-coupled bead pull-down assay. For the thermal shift assay [31], reactions were set-up in PCR tubes in a 20 μl volume containing 10 μM E-FABP and 10× SYPRO Orange dye (Invitrogen) in 20 mM HEPES pH 7 and 150 mM NaCl, containing either EI-05, BMS or DMSO control. PCR tubes were then sealed, centrifuged and heated from 25°C to 95°C at a rate of 1 degree/min on 7500 Real-Time PCR machine (Applied Biosystems). For the fluorescence assay, we measured the fluorescent signals of protein Trp as previously described [32]. Briefly, emission spectra of E-FABP Tyr/Trp and EI-05 were measured using Fluorolog-3 Spectrofluorometer (Horiba Scientific). Emission spectra were recorded from 300 to 500 nm in the presence and absence of E-FABP (0.5 μM) with addition of indicated concentrations of EI-05 or BMS-309413 (positive control) at 25°C.

### Generation of macrophages from bone marrow or peritoneum

For generation of GM-CSF-induced bone marrow-derived macrophages (GM-BMMs), bone marrow from mouse femurs or tibias was flushed with PBS, supplemented with 2% FBS. After lysis of red blood cells (R&D systems), the cells were plated in 100-mm tissue culture dishes with 5% FBS RPMI 1640 medium at 37°C/5% CO_2_ for 4 hours. Then the non-adherent cells were plated in 5% FBS RPMI 1640 medium with 20 ng/ml GM-CSF (R&D Systems). New 5% FBS RPMI 1640 medium with 20 ng/ml GM-CSF was added on day 2 and day 5. The cultured GM-BMMs were collected on day 7 for further experiments. For collecting peritoneal macrophages (PEMs), cells from peritoneum were flushed out with 10ml PBS and plated in 24-well plate for 2 hours. After wash off the non-attached cells, attached PEMs were used for experiments.

### Real-time PCR

For real-time PCR analyses, RNA was extracted from primary macrophages or macrophage cell lines using RNeasy Mini Kit (Qiagen). cDNA synthesis was performed with QuantiTect Reverse Transcription Kit (Qiagen). Quantitative PCR was performed with SYBR® Green PCR Master Mix using ABI 7500 Real-Time PCR Systems (Applied Biosystems). E-FABP, IFNβ, CXCL10, CXCL11, PPARγ, TNFα, IL-6, IL-10 and β-actin expression was analyzed by QuantiTect primer assays (Qiagen). Results were normalized to β-actin. Relative expression of the target genes was measured using the ΔΔCT approach.

### Confocal analysis

Macrophages from peripheral blood or peritoneum cultured on poly-D-lysine coated coverslips (NeuVitro) in a 24-well plate were treated with LPS (10 ng/ml) in the presence or absence of EI-05 (20 μM) for 24h. After fixation and permeabilization, the cells were stained with E-FABP specific antibody (R&D System). Confocal analysis was performed with Nikon Eclipse TE2000 confocal microscopy.

### ELISA

GM-BMMs or PEMs were stimulated with LPS (10 ng/ml) in the presence or absence of EI-05 for 24 hours. Culture supernatants were collected for measurement of protein levels of IFNβ with mouse ELISA kits (Biolegend) according to manufacturer's instructions.

### Western blot

To quantify the protein levels of E-FABP in macrophages, macrophages from peripheral blood or peritoneum with designated treatments were lysed in 1×lysis buffer with protease and phosphorylation inhibitors. Protein concentration was determined by BCA assay (Thermo Scientific). 10 μg of total protein was loaded. β-actin was quantified as a loading control. Mouse E-FABP antibody (goat) was from R&D Systems. Mouse β-actin antibodies (rabbit) were from Cell Signaling Technology.

### Flow cytometric analysis

Immune cells from peritoneum or tumors were subjected to surface staining or cultured with PMA (5 ng/ml; Sigma), ionomycin (500 ng/ml; Sigma) and Golgiplug (BD) for 6 hrs, harvested for surface and intracellular staining. Flow cytometric data were collected with a BD FACS Calibur™. Flow cytometric data analyses were performed with Flowjo (Tree Star). The following antibodies were used for cell staining: anti-CD4 (clone RM4-5), anti-CD8 (clone 53-6.7), anti-MHC class II (clone M5/114.15.2), anti-IFNγ (clone XMG1.2), anti-Granzyme B (clone GB11), anti-CD80 (clone 16-10A1) and anti-CD86 (clone GL-1), anti-CD11c (clone HL3) and anti-F4/80 (clone BM8).

### Mice and tumor model

C57BL/6 mice were bred and maintained in the animal facility at the Hormel Institute in accordance with the University of Minnesota Institutional Animal Care and Use Committee (IACUC). Six to ten-week-old female mice were fed standard chow diet and utilized for these experiments. All animal protocols were approved by IACUC in the University of Minnesota and followed national guidelines. E0771 cell line derived from a C57BL/6 mouse mammary adenocarcinoma was orthotopically injected into the mammary fat pad of the mice. EI-05 (10 mg/kg) or vehicle control were i.p. injected every other day until mice were sacrificed on day 27 after tumor cell injection. Tumors were measured at 3 day intervals with calipers at two bisecting diameters and an approximate volume was calculated by the formula (0.4) × (large diameter) × (small diameter)^2^ and single cells from tumor were prepared as previously described for analysis [8, 33].

### Statistical analysis

All quantitative data were shown as means ± SD. Unpaired, two-tailed Student's t-test was performed for comparison of results from different treatments. P value less than 0.05 is considered statistically significant.
